# Agreement between Five Experts and the Laguna ONhE Automatic Colourimetric Application Interpreting the Glaucomatous Aspect of the Optic Nerve

**DOI:** 10.3390/jcm12175485

**Published:** 2023-08-24

**Authors:** Carmen Mendez-Hernandez, Esperanza Gutierrez-Diaz, Marta Pazos, Rafael Gimenez-Gomez, Maria Dolores Pinazo-Duran

**Affiliations:** 1 Ophthalmology Department, Hospital Clinico San Carlos, Institute of Health Research (IdISSC), 28232 Madrid, Spain; 2 Department of Immunology, Ophthalmology and ORL IIORC, Complutense University of Madrid, 28232 Madrid, Spain; 3 Ophthalmology Department, Hospital 12 de Octubre, 28041 Madrid, Spain; espegd13@gmail.com; 4 Institut of Ophthalmology, Hospital Clinic Barcelona, Universitat de Barcelona, 08036 Barcelona, Spain; pazos@clinic.cat; 5 Ophthalmology Department, Hospital Universitario Reina Sofia, Instituto Maimonides de Investigación Médica, 14014 Cordoba, Spain; gimenezgomez@hotmail.com; 6 Ophthalmic Research Unit “Santiago Grisolia”, FISABIO and Department of Surgery, Medical School, University of Valencia, 46010 Valencia, Spain; dolores.pinazo@uv.es; 7 Spanish Network of Inflammatory Diseases REI-RICORS RD21/0002/0032, Institute of Health Carlos III, 28029 Madrid, Spain

**Keywords:** glaucoma, optic nerve head, deep learning, telemedicine, colourimetry, perfusion

## Abstract

Background: Optic nerve head (ONH) interpretation is a glaucoma screening method which may be influenced by criteria variability. Laguna ONhE software is a low-cost and non-invasive method of ONH analysis. Methods: We analysed the results of the Laguna ONhE application, interpreting 552 ONH images from the ACRIMA database, publicly available on the Internet, and compared them with the opinion of five experts. Diagnostic agreement was investigated using Cohen’s kappa (κ) with 95% confidence. Results: The kappa concordance index obtained with Laguna ONhE and the majority of the experts’ criterion (0.77) was significantly higher compared to that obtained with ACRIMA and the majority of the experts’ criterion (0.61). In 44.7% of the cases there was absolute agreement among the 5 experts and the Laguna ONhE program. Removing borderline cases from the analysis yielded increased diagnostic agreement (0.81). The area under the receiver operating characteristic (AUROC) of the Laguna ONhE program (0.953, *p* < 0.001) was not significantly different than AUROC of the majority of the experts’ criterion (0.925, *p* < 0.001), *p* = 0.052. Individually obtained expert’s AUROCs were significantly lower (0.636 to 0.913; *p* < 0.01). Conclusions: Laguna ONhE’s agreement with the experts is high, particularly where the diagnosis may be more obvious by the appearance of the ONH.

## 1. Introduction

Glaucoma is a group of pathologies affecting the optic nerve and the leading cause of irreversible blindness. Predictions estimated that this disease may affect 80 million people worldwide by 2020 and 111.8 million by 2040 [1]. Its prompt diagnosis is quite difficult owing to its almost asymptomatic onset; therefore, early detection of glaucoma is essential to prevent permanent vision loss. Digital fundus image evaluation has turned out to be an option for large-scale glaucoma screening given its convenience and relative affordability. Nonetheless, some of the problems with using fundus images for glaucoma diagnosis are subjectivity, low efficacy, and that experience and skills in the observer are essential. In addition, the process of image assessment is time-consuming and labor-intensive [2].

In 2019, a clinical database of images was published to help evaluate a diagnostic method for glaucoma using artificial intelligence (AI) [3]. The study was based on the ACRIMA database, an imaging database of 705 images obtained with TOPCON TRC retinography. Based on the opinion of two glaucoma experts, 396 of the images were classified as glaucomatous and 309 as normal ones.

The ACRIMA database’s images were graded by two glaucoma experts and no other clinical information was considered while labelling the images. As a result, the authors do not guarantee that the classification is correct and admit that the automatic procedure they designed has poor results against this classification, obtaining a poor area under the curve (AUROCs): AUROC = 0.7678 (95% confidence interval (CI) = 68.41–81.81%), with sensitivity a of 0.689 and specificity of 0.702.

The Laguna ONhE program is a colourimetric technique used to estimate the presence of haemoglobin and its effects on the optic disc [4,5,6]. In this way, the perfusion and morphology of the optic nerve can be screened using conventional colour retinography. Previous studies have demonstrated that this method has good sensitivity and specificity [7,8,9]. Additional deep learning methods concerning the segmentation of the optic nerve’s limit have been incorporated to facilitate its use and improve its reproducibility. For a better understanding of the Laguna ONhE method, we recommend reading Appendix A (Computing Development Setup), a scientific paper published by its authors [8].

Some differences between the authors’ methodology (ACRIMA) and the Laguna ONhE program are that ACRIMA automatically crops and resizes the optic disc screenings to a similar size. Hence, there is no quality control of the images and the size of the disc. On the contrary, the Laguna ONhE program identifies the position of the optic disc and segments it. Furthermore, if there are enough previous images of the same patient available, it compares the optic nerve size and takes it into account for the analysis of the case [10]. A final difference between ACRIMA and the Laguna ONhE program is that the latter analyses the quality of the image so that the operator can obtain a better one if needed.

The main purpose of this study is to evaluate the concordance of five glaucoma experts and the Laguna ONhE program in the analysis of images published on this ACRIMA database.

As a secondary objective, the diagnostic capability of the Laguna ONhE is evaluated against the consensus of the experts.

## 2. Materials and Methods

This was a cross-sectional study, approved by the Institutional Review Board of our university hospital, the Hospital Clinico San Carlos of Madrid, and carried out in accordance with the principles of the Declaration of Helsinki.

ACRIMA is a public database including glaucoma or normality labelled images that can be used for the evaluation of glaucoma classification methods. The images of this database derive from the ACRIMA project (TIN2013-46751-R) funded by the Ministry of Economy and Competitiveness of Spain, which aims for the development of automatic algorithms for retinal disease assessment.

The ACRIMA database is composed of 705 fundus images (396 glaucomatous and 309 normal) obtained from patients who have previously given their consent and collected in accordance with the ethical standards of the Declaration of Helsinki. All patients were selected based on their criteria and with no other clinical information by two glaucoma experts with at least fifteen years of experience. Fundus images from the ACRIMA database, focusing on the optic disc, were taken using the Topcon TRC retinal camera and IMAGEnet^®^ capture System. These series of images are public and the authors of this study were not involved in their production (https://figshare.com/articles/dataset/CNNs_for_Automatic_Glaucoma_Assessment_using_Fundus_Images_An_Extensive_Validation/7613135?file=14137700, accessed on 15 March 2019).

Firstly, the 705 images were evaluated using the Laguna ONhE program. The application has an automatic image quality assessment system which is applied before performing the optic nerve head colourimetric analysis. From this pre-quality analysis, 153 images were eliminated from the study.

**Figure 1:**
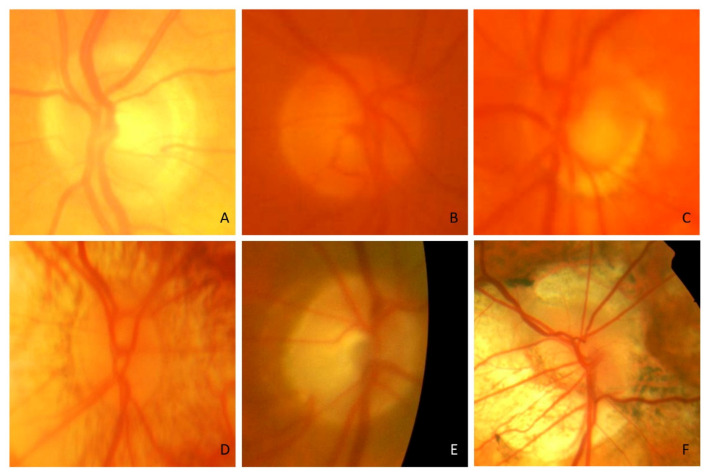
Examples of ACRIMA images excluded from this study. (**A**) red channel is saturated. (**B**,**C**) insufficient image quality (blurred optic nerve head image). (**D**) optic nerve head rim not clearly identified. (**E**) optic nerve head margin close to the edge of the image. (**F**) example of difficulties in segmenting the optic nerve head.

Figure 1 shows examples of images that were eliminated and the reason for exclusion. Among the excluded images, 16 of them were eliminated because the red channel was saturated (Figure 1A), 86 had insufficient image quality (blurred optic nerve head image, Figure 1B,C), in 4 images the optic nerve head rim could not be clearly identified (Figure 1D), and in 16 images the optic nerve head margin was very close to the edge of the image (Figure 1E) making it difficult to assess the optic nerve head and peripapillary rim area. Moreover, 31 images were not included owing to the optic nerve head morphology as significant difficulties were encountered in segmenting the optic nerve head (Figure 1F).

Overall, 552 optic nerve head images were suitable to analyse, 236 of which were normal and 316 glaucomatous, according to the original classification of the ACRIMA study.

The images were then analysed using the Laguna ONhE program, considering glaucomatous those which had a value of less than −15 on the Globin Discriminant Factor (GDF) index, the calculated cut-off value to achieve a specificity of 99% when identifying normal cases. In routine use when screening for Laguna ONhE, cases with a GDF value above 0 (specificity 95%) are considered normal, while borderline cases between the values of 0 and −15 are considered doubtful. In a first analysis, the doubtful images for Laguna ONhE were considered as normal and in a second analysis they were evaluated separately from those with higher diagnostic certainty.

At the same time, five experts in the diagnosis and treatment of glaucoma were asked to visually classify these cases as normal or glaucoma according to their criteria. The majority of the results were then calculated; i.e., the percentage of three or more concurring opinions. To evaluate the concordance between the five glaucoma experts and the Laguna ONhE program, an analysis of inter-rater agreement was performed using Cohen’s kappa (κ) with a 95% confidence interval.

Subsequently, Receiver Operating Characteristics (ROC) curves were assembled and Areas Under the ROC curves (AUROCs) were used to assess the diagnostic capacity of each procedure. AUROCS were compared using the DeLong method [11].

### 2.1. First Study

The agreement between the original ACRIMA classification, the five glaucoma experts, and the Laguna ONhE program were analysed. AUROCs were calculated by taking into consideration two different gold standards: the majority opinion of the experts and the results obtained using the Laguna ONhE program. In order to assess Laguna ONhE’s diagnostic capability, the majority opinion of the experts was considered as the gold standard. We evaluated the number of experts who agreed on the diagnosis of glaucoma considering the −15 limit for the Laguna ONhE GDF index as the gold standard.

### 2.2. Second Study

For the second calculation, the cut-off point GDF −15 was established as the gold standard. The optic nerve heads were classified as normal when the GDF index was >−15, and as pathological when the GDF value was <−15. Doubtful cases, i.e., those with a GDF index value between 0 and −15, were evaluated separately.

### 2.3. Statistical Analysis

The clinical statistical analyses were performed using the Excel 2016 program (Excel. Microsoft Corp., Redond, WA, USA) and MedCalc (Version 20.110-64 bits; MedCalc software bvba, Mariakerke, Belgium). Significant *p*-values were <0.05.

## 3. Results

Table 1 shows the proportion of cases with optic nerve heads identified as glaucomatous by the ACRIMA system, the Laguna ONhE program, and the five glaucoma experts. Expert 4 identified most cases as glaucoma, whereas expert 5 characterized as glaucoma the least number of cases.

### 3.1. First Study

Table 2 shows the Kappa value among the five experts, the ACRIMA data base, and the Laguna ONhE. Cases in the borderline situation for Laguna ONhE were classified as normal.

In summary, the agreement between the original ACRIMA classification, the experts, and Laguna ONhE can be considered moderate, and as substantial between Laguna ONhE and the majority opinion of the experts.

The Kappa index obtained using Laguna ONhE and the majority of the experts’ criterion (0.7686, CI: 0.71491–0.82233) was significantly higher compared to that obtained with ACRIMA and the majority of the experts’ criterion (0.6092, CI: 0.54640–0.67210).

**Figure 2:**
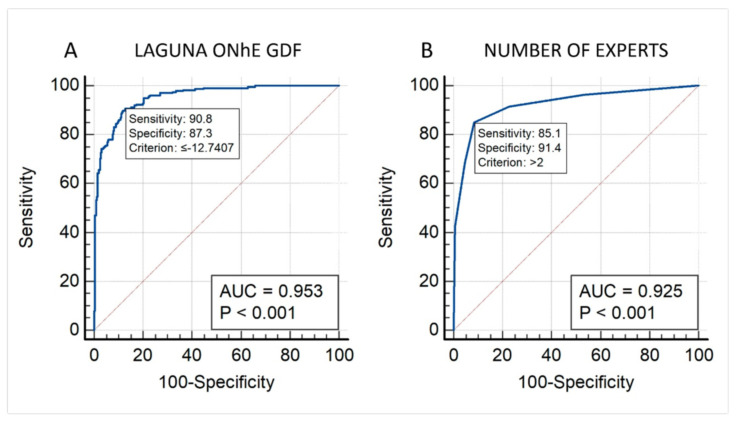
(**A**): AUROC obtained by the Laguna ONhE program considering the majority opinion of the experts as the gold standard. (**B**): Number of experts in agreement considering the −15 limit for the Laguna ONhE GDF as the gold standard.

Figure 2 shows the AUROC results obtained by the Laguna ONhE program considering the majority opinion of the experts as the gold standard (Figure 2A) and the AUROC obtained by the number of experts in agreement considering the −15 limit for the Laguna ONhE GDF as the gold standard, with the normal optic nerve head having a GDF index >−15, and pathological ones having a GDF values <−15. Considering Laguna ONhE the gold standard, 249 images were identified as normal and 303 as glaucomatous.

A comparison of ROC curves yielded a *p* = 0.052. Therefore, the Laguna ONhE criterion is not inferior to the majority opinion of the experts. However, it was significantly higher than that the ROC area obtained using the individual experts’ classification (Expert 1: 0.636, *p* < 0.0001; Expert 2: 0.770, *p* < 0.0001; Expert 3: 0.907, *p* < 0.0029; Expert 4: 0.885, *p* < 0.0001; Expert 5: 0.913, *p* < 0.0092).

**Figure 3:**
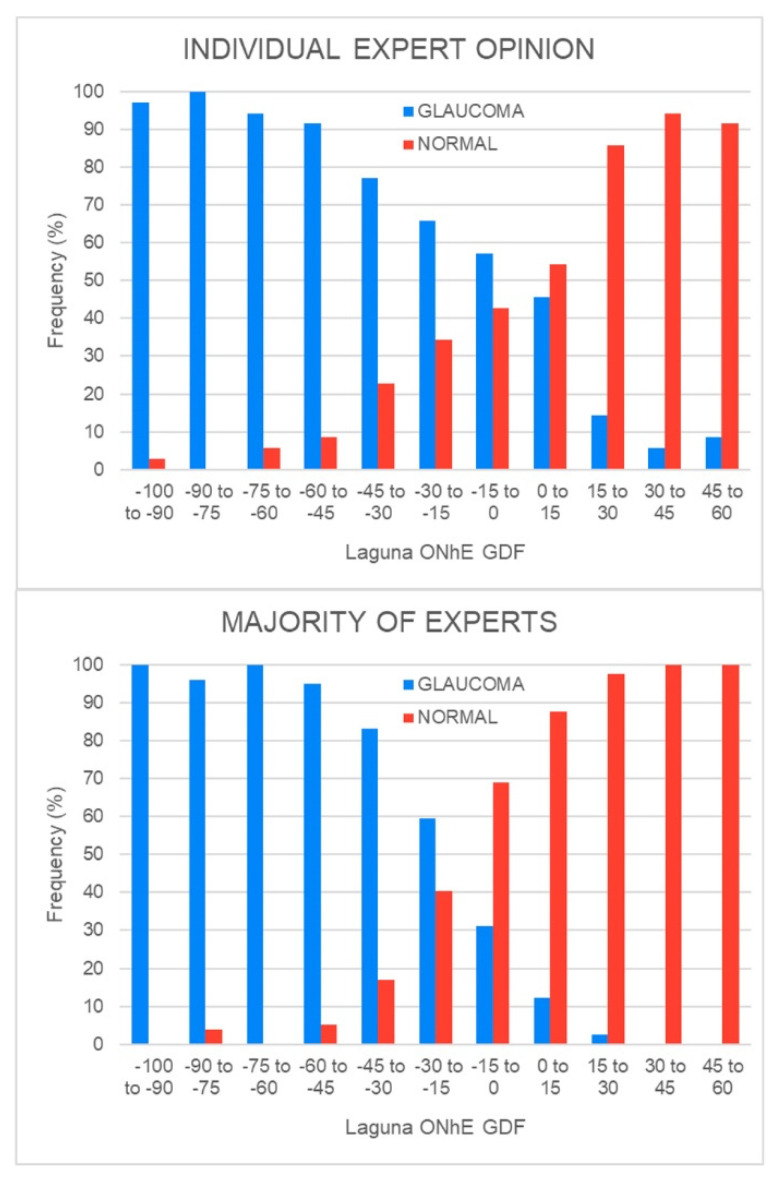
Frequency distribution of the experts’ individual and majority opinion on the value obtained by Laguna ONhE’s GDF index. Note that Laguna ONhE has a greater agreement with the majority opinion of the experts (**bottom** image) than with the individual experts (**top** image).

In 247 cases (44.77%), there was absolute agreement among the 5 experts and the Laguna ONhE (106 glaucoma and 141 normal cases), but majority opinion apparently discriminates better than individual experts (Figure 3).

**Figure 4:**
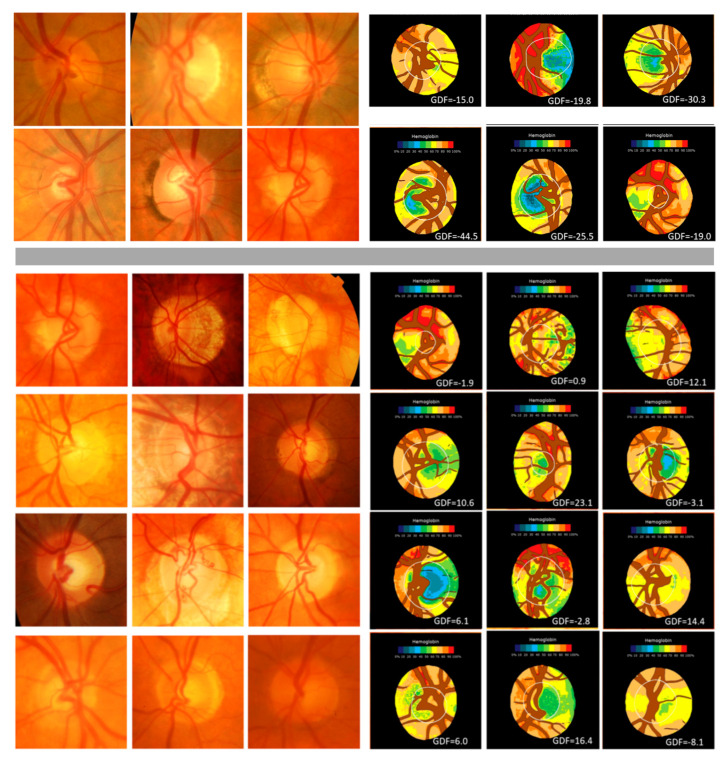
Main discrepant cases: in the upper 6 cases Laguna ONhE and two experts indicated normality, whilst ACRIMA and three experts diagnosed glaucoma. In the lower 12 cases, the opposite occurred.

The largest discrepancies were observed in 6 cases considered to be normal by ACRIMA and three experts, whilst Laguna ONhE and two of the five experts categorized them as glaucoma (six upper optic nerve head images in Figure 4). Moreover, 12 cases were considered glaucoma by ACRIMA and three experts but were thought to be normal by Laguna ONhE and the other two experts (12 lower optic nerve head images in Figure 4).

### 3.2. Second Study

It is also important to analyse the borderline cases separately from those with a more obvious diagnosis, as many images in the ACRIMA series are not easy to interpret visually.

Out of the 552 cases in the study, 258 had GDF values above 0. This is the theoretical limit defined by the method for a specificity of 95% and indicated in the screening application in green. On the other hand, 249 had a GDF lower than −15 which is the theoretical limit for 99% specificity and presented in red. That is, 45 images were considered doubtful while, for the other 507 images, the diagnosis was more obvious.

The diagnostic agreement of Laguna ONhE with the majority opinion of the five experts in these 507 cases was kappa = 0.81, (CI = 0.76–0.86) which is considered “Almost perfect agreement”. Particularly relevant is the fact that, when the GDF was above 0, the majority of experts’ criterion indicated normality in 95.3% of the cases, in line with the theoretical specificity of 95% with which this cut-off has been designed.

Table 3 shows the kappa values obtained between the original ACRIMA classification and the experts’ opinion in the remaining 45 cases (8.2% of the total) that were considered by Laguna’s ONhE as doubtful by having an GDF between −15 and 0. When assessing these images, the agreement between the five experts was very low with a mean kappa = 0.18376 (Slight agreement); although if we estimated the agreement of each of them using the majority criterion, we obtained a kappa of 0.47 (Moderate agreement).

## 4. Discussion

Achieving a completely reliable gold standard for the diagnosis of glaucoma is virtually impossible and continues to be a cause for debate [12]. There are many studies that are based on a subjective visual classification of the optic nerve, such as the ACRIMA classification which employs the subjective opinion of the examiner as the gold standard in decision making. Several artificial intelligence programs are also based on these subjective classifications, meaning that they consider only expert assessment of the optic nerve [13,14].

If the subjective interpretation of the optic disc stated by an expert was a sufficient criterion, other measurements such as the visual field, the optic nerve fibre layers thickness using optical coherence tomography (OCT), or the analysis of the optic disc with colourimetry using programs like Laguna ONhE would not be needed. However, expert opinion does not seem to be enough to reliably establish glaucoma diagnosis and, in any case, the sensitivity and specificity of this criterion are not always optimal in addressing the issue.

Laguna ONhE is a method for the colourimetric evaluation of the distribution of haemoglobin at the optic nerve head. By colorimetry, the nerve tissue is compared with the vessels of the central retinal artery and vein branches which are used as a standard or reference value. This allows for the estimation of the presence of haemoglobin in each area of the optic disc. The distribution of haemoglobin in the inferior and superior zones of the nerve in relation to the nasal and temporal zones, and the relationships between the size and shape of the cupping, weighted according to the size of the optic disc, as well as other factors such as the presence of peripapillary atrophy provide the globin or glaucoma discriminant function, the GDF index, in which the deep learning glaucoma vs. normal classifier has a high influence, with an approximate range of −100 to +85 units [15].

The application is fully automated using several AI mechanisms, including deep learning convolutional neural networks, to determine the quality of the image, segment the limits of the optic nerve head, identify its laterality, establish the presence or absence of the entire optic disc and its surroundings, achieve the segmentation of its vessels, and, finally, identify the existence of normal or glaucoma characteristics [8]. Other algorithms are responsible for recognizing whether the image has been zoomed in and estimating the disc area, the area of the cup, and the area of the rim sectors [10].

AI and convolutional neural networks have been used in image recognition. Some recent studies use optic nerve head imaging, developing an objective machine learning classification pattern for glaucomatous optic disc classification [16,17,18]. In a study in which images from 163 eyes, assessed by glaucoma specialists and acquired using OCT, were used, a total of 91 parameters that included basic information about the patients’ eyes were selected. Neural automatic classification models were built using neural networks to create classification models. The accuracy of such networks used was 87% [19]. Other recent studies using the fundus image and deep learning, as well as segmented optic disc images, have proposed convulsive neural networks to increase the efficiency of the extraction module and different networks for the optic nerve head and optic nerve cup. Jiang et al. used boxes to trace the limits of the optic nerve head and the optical cup, with the latter being inscribed as ellipses. Their convolutional neural network, based on segmentation of optical discs and cups, showed small overlap errors on the optical disc and cup (6.3% to 20%) which could be useful in the early detection of glaucoma [20].

By analysing 1542 retinographies using the convulsive neural network, Deep Convolution Network and Resnet, another study conducted by Ahn et al. correctly detected both early and advanced glaucoma with an accuracy of 92.2% [21].

The Laguna ONhE program is commercially available and has been applied in glaucoma screening in optometry and ophthalmology centres mainly in Central Europe and Scandinavia. It has been used since 2013 [4] in the study of different forms of chronic glaucoma, mainly, but also congenital glaucoma.

The application has been compared with other diagnostic methods such as OCT and angio-OCT. Its usefulness in the diagnosis of early glaucoma and its power associating its information with the irregularity of the visual field have been demonstrated [7,8,22]. Nonetheless, it would be interesting to carry out comparative studies with other methods using AI and the Laguna ONhE programme.

Confirming that a patient has glaucoma is highly complex. It is believed that half of glaucoma patients remain undiagnosed and many other patients with glaucoma are incorrectly diagnosed [23]. In the UK, for example, between 20% and 65% of glaucoma patients are misdiagnosed. The health and economic consequences of this are highly relevant [24].

The question to be considered is as follows: which criteria should be taken into account to efficiently identify such a prevalent disease in order to correctly diagnose a glaucoma patient?

The most important criteria for a glaucoma screening procedure are that the method should be both fast and cost-effective so as many people as possible can be screened. Additionally, the method should be very specific in order to not overburden the health care system. As glaucoma is generally a slowly progressing disease, it is preferable to choose a procedure with high specificity rather than one with high sensitivity. By repeated application of the screening method, previously undetected cases could be distinguished before advanced stages of the disease are reached. If such a screening approach was not chosen, mass diagnoses would be unfeasible owing to saturation of the health care system. It should also be considered that diagnostic confirmation requires demonstrating the worsening of the patient’s condition, even within the limits of normality, and in these cases an isolated examination is not enough. Therefore, the chosen method should allow for proof of the progression of the disease.

Intraocular pressure (IOP) is the main risk factor for developing glaucoma. However, IOP measurement cannot be the criterion for mass screening because it bypasses low-tension glaucoma. Furthermore, it requires training the examiners and the use of devices that can only be legally used by ophthalmologists and optometrists. Moreover, corneal thickness is a confounding factor that affects the results. As a result, the above factors make this method an unfeasible initial diagnosing criterion.

Visual field testing is also not an adequate option because it requires complex instruments that are not always available and qualified personnel. In addition, since this method has low specificity, it requires good patient cooperation or else several training tests are needed until the learning effect is ruled out. Even if equipment to perform perimetry becomes cheaper, for example, by using virtual reality equipment, its mass application with high specificity is unattainable.

Of course, more sophisticated techniques, such as OCT, are not available to every ophthalmological clinic because of their cost, the technical difficulties, and the complex interpretation of the images.

The observation of a suspicious optic disc could be a suitable initial evaluation if experts competent to interpret the retinography images and establish a consensus diagnostic criterion were available everywhere. The latter method is not perfect because its specificity does not exceed 90%, meaning that many subjects would have to be revaluated. It also requires glaucoma experts with extensive experience which is impossible since they are not always available for mass screening of the population.

Moreover, the results may be limited and variable depending on level of expertise. Our results, obtained by glaucoma experts with extensive experience, are among the most concordant of all published studies [25,26,27,28,29,30,31,32,33], although in some cases, the aim was not a diagnostic evaluation but merely to estimate the morphological characteristics of the optic disc [34].

The unlikelihood of having available experts for mass evaluations is itself enough to exclude this as a population-wide method. Instead, a system that is easy to use, automatic, cheap, fast, with high specificity, and with a sensitivity at least equivalent to the best expert or a pool of experts, could help with the establishment of mass screening.

The Laguna ONhE program can be used on optic nerve head images, obtained using simple fundus cameras, with minimal training. If a retinography network system, such as the ones used in retinal monitoring of diabetic patients is also available, the feasibility of mass population screening is even greater.

The level of specificity of Laguna ONhE to identify glaucoma cases (GDF < −15) is 99%. This has been verified in several previous studies and is currently being verified in retrospective and prospective studies of diabetics [15]. On the contrary, the specificity of an expert’s optic nerve assessment is rather low, being estimated 47% for residents, 53% for optometrists, and 60% for general ophthalmologists [35]. Our expert agreement results are consistent with those of previous studies.

From the above, it can be deduced that the balance between sensitivity and specificity in the Laguna ONhE program is superior from that of isolated experts. Indeed, we have found that the Laguna ONhE results are similar to the majority opinion of experts, and also exceeds it when other less subjective information is taken into account. The Laguna ONhE program offers quantitative data not only related to the morphology of the nerve [36], but also to its vascularization [22,37], presenting high sensitivity and precocity [38], as shown in past studies.

Additionally, it allows images to be monitored which can lead to a diagnosis if significant changes, even at the limits of normality, are observed, and to compare the changes throughout the follow-up period [15].

The Laguna ONhE program could be used as a large-scale glaucoma screening method in optometry and ophthalmology centres.

Those patients suspected of having glaucoma should be referred to ophthalmology clinics to complete the ophthalmological examination with intraocular pressure and pachymetry measurements, retinal nerve fibre layer analysis using SD-OCT, and perimetry. Thus, automated retinography analysis using the Laguna ONhE program would be the first step in the diagnosis of glaucoma.

It should be emphasized that Laguna ONhE program should only be applied for glaucoma screening to identify glaucomatous changes in the ONH. To confirm the diagnosis, it would also be necessary to perform applanation tonometry applying a correction factor according to the central corneal thickness, daily curve of intraocular pressure, and optic nerve fibre layer measurements using SD-OCT to determine a diagnosis of pre-perimetric glaucoma, as well as computerised perimetry in already established functional damage.

## 5. Conclusions

In summary, Laguna ONhE’s agreement with experts on the assessment of the optic nerve head is high. Its diagnostic classification agrees with that of the majority of experts in cases where the diagnosis may be made more obvious by the appearance of the optic nerve head; in cases where Laguna ONhE classifies as doubtful, the agreement between experts is low. Therefore, the Laguna ONhE program is an automated optic nerve head assessment system that could be useful in glaucoma screening and shows high agreement with the assessment of the glaucoma experts.

Retinography analysis in primary care centres, ophthalmological clinics, and optometric centres can be an effective glaucoma screening system owing to its high specificity. Despite the need for future studies implementing the Laguna ONhE application in combination with a retinography network system, perimetry, and intraocular pressure measurement, it may eventuate as a mass glaucoma screening system, particularly considering its current specificity and cost-effectiveness.

## Figures and Tables

**Table 1:** Proportion of optic nerve heads identified as glaucomatous by the experts and the Laguna ONhE program.

	% GLAUCOMA
ACRIMA	57.2
Laguna ONhE GDF	45.1
EXPERT 1	43.7
EXPERT 2	48.4
EXPERT 3	46.4
EXPERT 4	57.4
EXPERT 5	27.0
MAJORITY	43.1

**Table 2:** Kappa values in the full sample.

Kappa	ACRIMA	Laguna ONhE GDF	EXPERT 1	EXPERT 2	EXPERT 3	EXPERT 4	EXPERT 5
Laguna ONhE GDF	0.58201						
EXPERT 1	0.58374	0.72118					
EXPERT 2	0.32329	0.41305	0.41957				
EXPERT 3	0.57691	0.67523	0.74768	0.37506			
EXPERT 4	0.55935	0.61430	0.60882	0.37541	0.60211		
EXPERT 5	0.35803	0.53714	0.59214	0.34525	0.53898	0.37599	
Average	0.49722	0.59218	0.51167				
*t* test	ACRIMA	Laguna ONhE GDF					
Laguna ONhE GDF	0.11566						
ALL EXPERTS	0.41766	0.14364					
Majority	0.60925	0.76862	0.87831	0.46499	0.84629	0.69110	0.62478

**Table 3:** Kappa values in borderline cases for the Laguna ONhE GDF index.

	ACRIMA	EXPERT 1	EXPERT 2	EXPERT 3	EXPERT 4	EXPERT 5	
EXPERT 1	0.18957						
EXPERT 2	−0.09091	0.13223					
EXPERT 3	0.34783	0.42308	−0.06838				
EXPERT 4	0.23881	0.30636	−0.03604	0.47368			
EXPERT 5	0.09174	0.33912	−0.01563	0.32990	0.19263		
Average	0.26870	0.18376					
							Average
Majority	0.27488	0.68894	0.13223	0.71154	0.56647	0.45928	0.47222

## Data Availability

The datasets analysed and generated during the study are available upon request.
